# Improving the photocatalytic reduction of CO_2_ to CO for TiO_2_ hollow spheres through hybridization with a cobalt complex†

**DOI:** 10.1039/c8ra03211d

**Published:** 2018-06-05

**Authors:** Jinliang Lin, Xiaoxiang Sun, Biao Qin, Ting Yu

**Affiliations:** Department of Chemical and Engineering, Zunyi Normal College 563000 Zunyi P. R. China jinliang_lin@163.com +86-851-28927159 +86-851-28927159; Department of Chemical and Engineering, Qiannan Normal University for Nationalities 558000 DuYun P. R. China

## Abstract

A chemical system with enhanced efficiency for electron generation and transfer was constructed by the integration of TiO_2_ hollow spheres with [Co(bipy)_3_]^2+^. The introduction of [Co(bipy)_3_]^2+^ remarkably enhances the photocatalytic activity of pristine semiconductor photocatalysts for heterogeneous CO_2_ conversion, which is attributable to the acceleration of charge separation. Of particular interest is that the excellent photocatalytic activity of the heterogeneous catalysts can be utilised for a universal photocatalytic CO_2_ reduction system. Yields of 16.8 μmol CO and 6.6 μmol H_2_ can be obtained after 2 h of the photoredox reaction, and the apparent overall quantum yield was estimated to be 0.66% under irradiation at *λ* = 365 nm. The present findings clearly demonstrate that the integration of electron mediators with semiconductors is a feasible process for the design and development of efficient photochemical systems for CO_2_ conversion.

## Abstract 2

A chemical system with enhanced efficiency for electron generation and transfer was constructed by the integration of TiO_2_ hollow spheres with [Co(bipy)_3_]^2+^.

## Graphical abstract

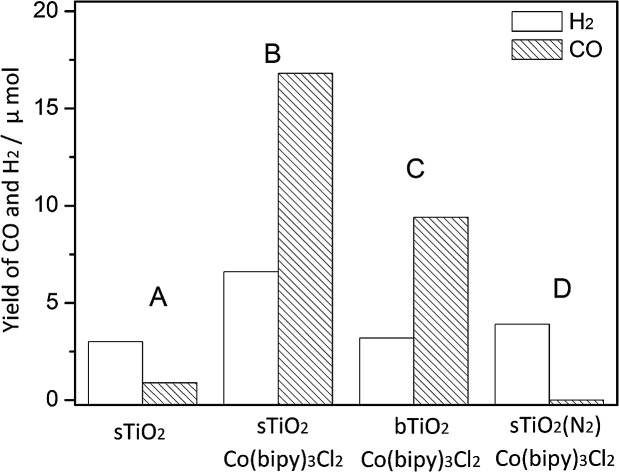

## 1 Introduction

The photochemical reduction of CO_2_, as a model reaction of artificial photosynthesis, has obtained much attention because it may provide solutions for overcoming both the problem of global warming and the shortage of fossil resources.^1^ Of particular interest is the conversion of CO_2_ into the energy carrier CO, which is an important chemical feedstock used to form syngas in combination with H_2_, and can be used to replace the steam reforming of fossil fuels in the petrochemical industry.^2^

Photoelectrocatalytic CO_2_ reduction has been achieved on various kinds of material, including inorganic semiconductors, carbon based semiconductors, metal complexes, supermolecules, and their derivatives.^3^ Of these, TiO_2_ is one of the most well-known and widely used materials due to its prominent properties, namely that it is non-toxic, low cost, chemically stable, and exhibits good radiosity.^4^ In order to improve optical performance, it has been combined with various metals or other semiconductors to form hybridized composites in an attempt to reduce the band gap or suppress the recombination of photogenerated charge carriers.^5^ Another way to achieve desirable photocatalytic behavior is by preparing catalytic materials that possess special structures. Hierarchical structured TiO_2_ with high surface areas such as hollow spheres has been designed for the purpose of increasing active site exposure. The beneficial effects of particle morphology on photochemistry have also been proven, with previous examples investigating geometric effects and electronic nature.^6^ Additionally, previous works have proven that spherical morphology endowed Ti-base materials exhibit improved photocatalytic performances.^7^

Furthermore, hollow sphere structured TiO_2_ can be further modified through hybridization or ion-doping strategies, and exhibits an enhanced photocatalytic performance for CO_2_ activation. Fang *et al.* has presented novel Ti-based spherical photocatalysts with a large mesoporous volume and hierarchical porosity.^8^ These exhibited an improvement in photocatalytic activity and an enhancement in the efficiency for the solar reduction of CO_2_. Additionally, a one-pot template-free method has been reported for the preparation of Cu(ii) incorporated TiO_2_ hollow microspheres for photo-driven CO_2_ reduction. The effect of metal vacancies on the physicochemical properties has been proven to be critically important for metal oxides.^9^ Hollow spheres consisting of alternating titania nanosheets (Ti_0.91_O_2_) and graphene nanosheets have been prepared by Zou’s group.^10^ There was a nine-fold enhancement of the photocatalytic activity on such hollow spherical materials compared to commercial P25 for photocatalytic CO_2_ conversion. They have also reported a Au–TiO_2_ nanocomposite with bifunctional linker molecules which was applied for the photocatalytic reduction of CO_2_ into hydrocarbon fuels.^11^ Several carbon fuels were produced over the Au–TiO_2_ nanocomposite, including CO, CH_4_, CH_3_OH and CH_3_CH_2_OH, under different forms of light irradiation and in different reaction systems. A Cu_2_O/TiO_2_ composite exhibited higher efficiency in the photocatalytic reduction of CO_2_ into CH_4_ under visible-light irradiation (*λ* ≥ 420 nm).^12^ This is because of the formation of a p–n heterojunction in the composites, resulting in the efficient suppression of the recombination of the photogenerated electrons and holes as well as an improved stability of the catalyst and, accordingly, an improved visible-light photocatalytic activity. Additionally, Jiang *et al.* found that a significantly improved photocatalytic activity obtained by hybrid carbon@TiO_2_ hollow spheres was mainly due to the increased specific surface area, CO_2_ uptake, local photothermal effect, enhancement of light absorption and charge transfer efficiency.^5*b*^ A comprehensive review on surface and interface design in cocatalysts concerning CO_2_ reduction has been presented by Bai *et al.*^13^ As discussed, the rational material design of cocatalysts would be an effective route for pursuing their maximum contribution to the performance of photocatalysts.

The strategy of hybrid photocatalysts is a promising approach for the rational design of efficient and stable artificial photosynthetic systems. This is because homogeneous processes exhibit high quantum efficiencies, while heterogeneous processes possess desirable stabilities. Z-scheme water splitting systems, redox couples (*e.g.*, Fe^3+^/Fe^2+^, IO^3−^/I^−^, [Co(bipy)_3_]^3+/2+^ and [Co(phen)_3_]^3+/2+^) and reduced grapheme oxides have been revealed to act as electron mediators to deliver photogenerated electrons from the O_2_-evolving catalyst to the H_2_-evolving catalyst in the two-photon type photocatalytic system.^14^ Also, various metal complexes, which mostly can facilitate charge separation and lower the energy barrier, are usually employed as co-catalysts for CO_2_ conversion. In earlier years, a high-efficiency photocatalytic CO_2_ reduction system consisting of semiconductor powders and a Ru-based complex was reported by Bhatt and coworkers.^15^ Xu *et al.* have developed an efficient hybrid CO_2_ photoreduction system by mixing CdS nanoparticles and CoCl_2_/bipy in an acetonitrile solution, which affords a high apparent quantum yield of 1.0% for CO formation under monochromatic irradiation (*λ* = 470 nm).^16^ Our previous work revealed that a similar reaction mechanism can be further extended to both heterogeneous and homogenous photocatalytic CO_2_ reduction systems in acetonitrile solution.^17^ More recently, Kuriki *et al.* has successfully developed several types of hybrid photocatalyst consisting of semiconductors and metal complexes, which have both the efficient CO_2_ reduction abilities supplied by the metal-complex unit and the strong oxidation power of semiconductors.^18^

Complex/semiconductor hybrid photocatalysts represent a feasible approach to achieving high efficiency semiconductors for solar fuel production. However, the research in this direction is still in an early stage and therefore leaves much room for further development. In this paper we present the synthesis of spherical TiO_2_ (denoted as “sTiO_2_”), and then employ it as photocatalyst for CO_2_ reduction under UV-light irradiation. Based on the analysis, it is reasonable to think that the hybrid of TiO_2_ hollow sphere and homogeneous methods may be an ideal pathway towards high efficiency. The spherical TiO_2_ with higher surface area exhibits a superior photocatalytic performance to bulk TiO_2_ (denoted as “bTiO_2_”). More importantly, this study also shows that the combination of the sTiO_2_ catalyzed photosystem with [Co(bipy)_3_]^2+^ as a cobalt complex redox shuttle induces a highly efficient and selective electron transfer for the reduction of CO_2_. The reaction activity in the presence/absence of cobalt complex shows that this is a feasible way to obtain high efficiency photocatalytic CO_2_ conversion.

## 2 Experimental

### 2.1 Chemicals

All reagents were commercially provided and used without further purification. Diethylenetriamine (DETA, 97%), titanium(iv) isopropoxide (TIP, 97%) and 2,2-bipyridine (bipy, 98%) were purchased from Alfa. Isopropyl alcohol (IPA, 98%), acetonitrile (MeCN, AR), triethanolamine (TEOA, AR) and cobalt chloride hexahydrate (CoCl_2_·6H_2_O, 99.9%) were purchased from China Sinopharm Chemical Reagent Co. Ltd.

### 2.2 Preparation of TiO_2_ samples

The TiO_2_ spheres were prepared using a hydrothermal method as described in previous reports:^19^ 0.05 mL of DETA was added to 75 mL of isopropyl alcohol, and the mixture was stirred for 5 minutes. Then, 2.5 mL of titanium(iv) isopropoxide was added. The reaction solution was then transferred to a 100 mL Teflon-lined stainless steel autoclave and the temperature was maintained at 180 °C for 24 h. The autoclave was then taken out of the oven and left to cool naturally to room temperature. The puce products were isolated *via* centrifugation, cleaned with four cycles of centrifugation and washing in ethanol and water respectively, and dried at 60 °C overnight. All of the products were calcined at 450 °C for 2 h with a heating rate of 1 °C min^−1^ to obtain a highly crystalline anatase phase. The bulk TiO_2_ was directly obtained through hydrolysis: 5 mL of titanium(iv) isopropoxide was added to 95 mL of water, and the mixture was stirred for 10 minutes. The following processes, including isolation and calcination, were the same as those for the TiO_2_ sphere.

### 2.3 Characterization

X-ray diffraction (XRD) patterns were collected using a Bruker D8 Advance X-ray diffractometer (Cu Kα irradiation, *λ* = 1.5406 Å), irradiating with a scanning rate of 0.02 deg s^−1^. A transmission electron microscope (TEM, JEOL-200CX) was used to observe the morphology of the particles. The scanning electron microscope (SEM, FEI-Philips CM-20) images were obtained with an electron microscope coupled to an energy-dispersive X-ray spectrometer (EDS) with an accelerating voltage of 200 kV. The Brunauer–Emmett–Teller (BET) specific surface areas of initially treated samples at 453 K for 8 h were calculated from the nitrogen adsorption–desorption isotherms obtained at 77 K with the Micromeritics ASAP 2020 system. Ultraviolet and visible absorption spectra were recorded at room temperature with a UV-vis (Varian Cary 5000) spectrophotometer. The detection of the Co^I^ transition was conducted in a sealed container adapted to accommodate the recording equipment, and the sample was exposed to light irradiation for 10 min before the test. Photoluminescence (PL) spectra were observed with a spectrophotometer (Hitachi, F-4600), with an excitation wavelength of 400 nm. The electrochemical behavior was tested on a CHI660E workstation (Shanghai Chenhua Instruments, China) using a three-electrode electrochemical cell with a working electrode, a platinum counter electrode and a saturated calomel electrode (SCE) as a reference electrode in a 0.1 M KCl aqueous solution. The working electrode was made of indium-tin oxide (ITO) glass.

### 2.4 Photocatalysis test

All experiments were performed in a Schlenk flask (80 mL) under an atmospheric pressure of CO_2_ (1 atm).^20^ In the Schlenk flask, TiO_2_ (50 mg), bipy (15 mg) and CoCl_2_ (50 μmol) were added to a mixture of solvent (5 mL) and TEOA (1 mL). The system was subjected to vacuum degassing and backfilling with pure CO_2_ gas. This process was repeated three times and after the last cycle the flask was back filled with CO_2_. Then the system was irradiated with five non-focused 6 W UV lights (Hitachi F6T5, 365 nm) with vigorous stirring at 20 °C controlled by a water-cooling system. The produced gases (CO, H_2_) were detected by a gas chromatograph (Agilent 7890B, Agilent Technologies) equipped with a packed column (TDX-1 mesh 42/10). Ar was used as the carrier gas. The apparent quantum yield (AQY) for CO/H_2_ generation was measured using the same photochemical experimental setup. The intensity of light irradiation was measured as 2.6 mW cm^−2^ (CEAulight, AULTT-P4000) and the irradiated area was 1.0 cm^2^.

## 3 Results and discussion

**Fig. 1:**
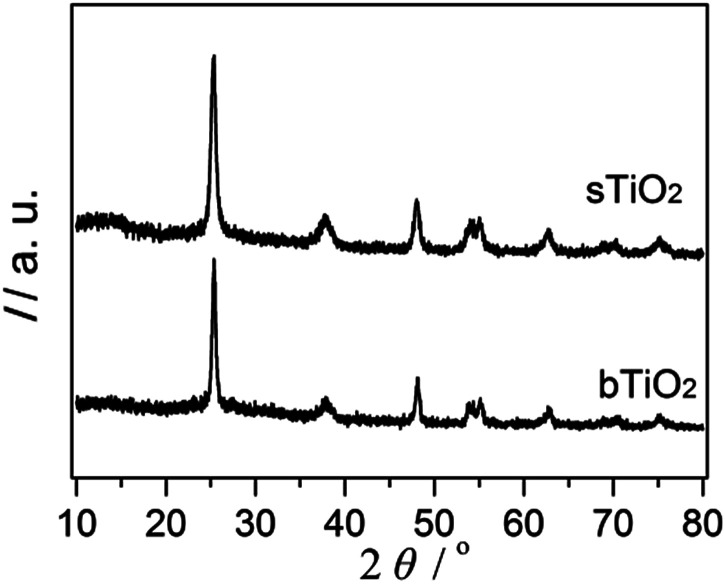
XRD spectra of the as-prepared sTiO_2_ and bTiO_2_.

The high crystallinity and phase purity of the spherical resultant material (sTiO_2_) were confirmed *via* XRD (Fig. 1). The diffraction peaks of TiO_2_ were observed at 2*θ* = 25.1° (101), 37.6° (004), 47.9° (200), 53.7° (105), 54.5° (211) and 62.7° (204), which agrees with the corresponding crystal planes of anatase TiO_2_ (JCPDS file no. 21-1272). Additionally, the TiO_2_ directly obtained *via* hydrolysis and then calcination at 450 °C that was used as a reference sample (bTiO_2_) exhibited a pattern corresponding to the anatase phase, which is the same as the spherical sample.

**Fig. 2:**
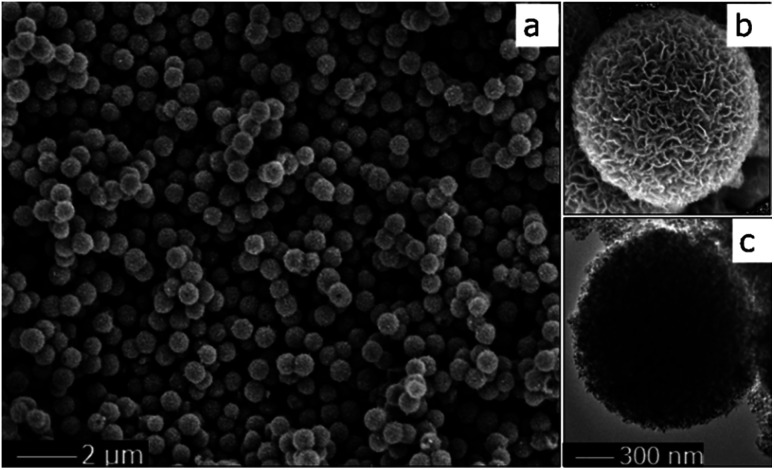
SEM (a and b) and TEM (c) images of the TiO_2_ spheres.

The morphology of the TiO_2_ sphere was verified *via* SEM and TEM. As shown in Fig. 2a, the SEM images display that the as-synthesized TiO_2_ structures are typically spherical in shape. Fig. 2b indicates that the units of the spheres are constructed with nanosheets. These plates with interlaced structure are also revealed in the SEM image. The superstructure was further confirmed *via* TEM (Fig. 2c), and is in accord with previous reports.^19^ These results indicate that the three-dimensional structure with pieces supporting each other greatly contributes to the high surface area and the stability of the catalytic material during the photochemical reaction.

**Fig. 3:**
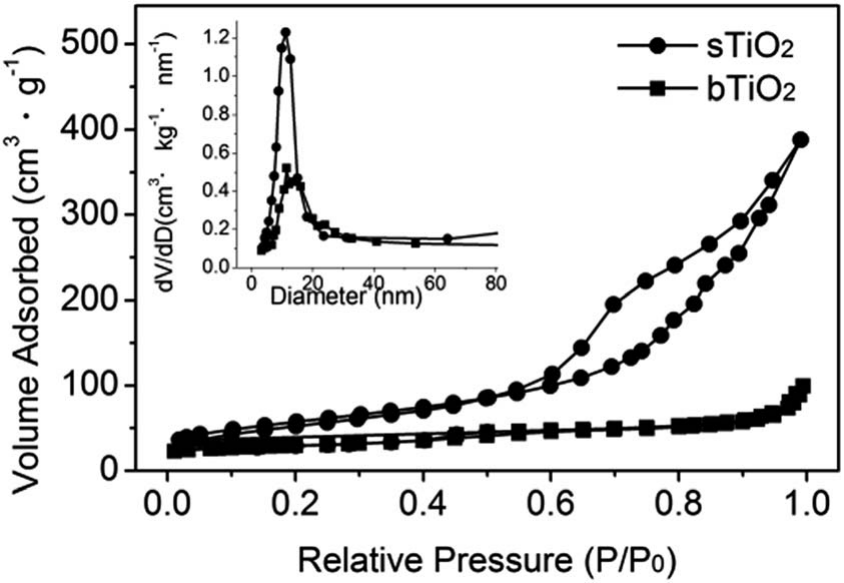
N_2_ adsorption/desorption isotherm of the as-prepared spherical TiO_2_ (●) and bulk TiO_2_ (■). The inset shows the pore size distributions calculated using the BJH method.

The self-assembly of the hollow structured TiO_2_ produces a nanoporous structure, as confirmed by the N_2_ adsorption/desorption isotherm shown in Fig. 3. It gives a type-IV isotherm with a type H_3_ hysteresis loop, which is indicative of a mesoporous structure.^21^ The relatively narrow pore size distribution of sTiO_2_ (Fig. 3, inset) calculated using the Barrett–Joyner–Halenda (BJH) method from the two branches of the isotherm signifies that most of the pores have sizes in the range 5–15 nm. Such a porous structure gives rise to a surface area of 136 m^2^ g^−1^, as calculated *via* the Brunauer–Emmett–Teller (BET) method. Obviously, as demonstrated in Fig. 3, the surface area of sTiO_2_ is much higher than that of bTiO_2_ (*ca.* 14 m^2^ g^−1^).

**Fig. 4:**
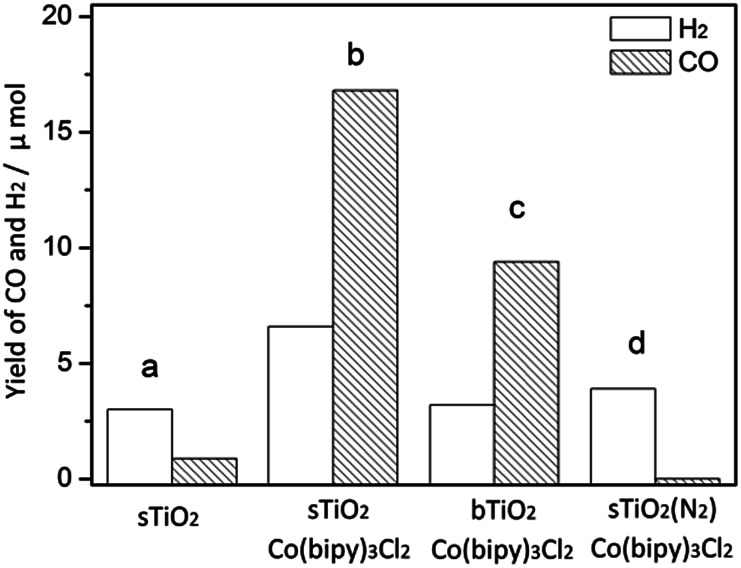
The photocatalytic performance of (a) sTiO_2_, (b) sTiO_2_ + Co(bipy)_3_Cl_2_, (c) bTiO_2_ + Co(bipy)_3_Cl_2_ and (d) sTiO_2_ + Co(bipy)_3_Cl_2_ under an N_2_ atmosphere.

As shown in Fig. 4, only a trace amount of CO (<1 μmol) was detected in the current system, and the yield of H_2_ was also moderate (3 μmol). When Co(bipy)_3_^2+^ was present as an effective electron transport carrier, as expected, the reaction was promoted dramatically, yielding nearly a 20-fold increase in CO (16.8 μmol) and twice the amount of H_2_ (6.6 μmol). The apparent overall quantum yield of this CO_2_ photoreduction system was estimated to be 0.66% under irradiation at *λ* = 365 nm. The production of CO and H_2_ on bTiO_2_ was 9.4 μmol and 3.2 μmol respectively, in spite of this system also containing [Co(bipy)_3_]Cl_2_. We have also tested the photocatalytic performance on P25 (Degussa) as a standard reference, which leads to the formation of CO (19.3 μmol) and H_2_ (8.9 μmol) in the current system. This is mainly attributable to its intrinsic structure being a 70% rutile phase and 30% anatase phase titanium mixture. CO production on sTiO_2_ is 2.5 μmol less than that on P25. When CO_2_ was replaced by N_2_ in the system, evidence was observed that confirmed the participation of CO_2_ in the reaction, because only H_2_ gas (3.9 μmol) was detected under these reaction conditions, and no CO was detected. This result confirmed that the carbon source of CO production is CO_2_, and not other organic ingredients. Additionally, control experiments showed that there was no CO generation in the absence of light or TiO_2_. In addition, energy-dispersive X-ray spectroscopy (EDS) analysis and elemental mapping are shown in Fig. S1 to S6†, and were used to determine the Co content in bTiO_2_, sTiO_2_ and sTiO_2_ samples after reaction. There was no change detected in the Co content in sTiO_2_ after the reaction, compared with the samples before the reaction. The results indicate the existence of the Co-complex in this system throughout the studied process.

**Fig. 5:**
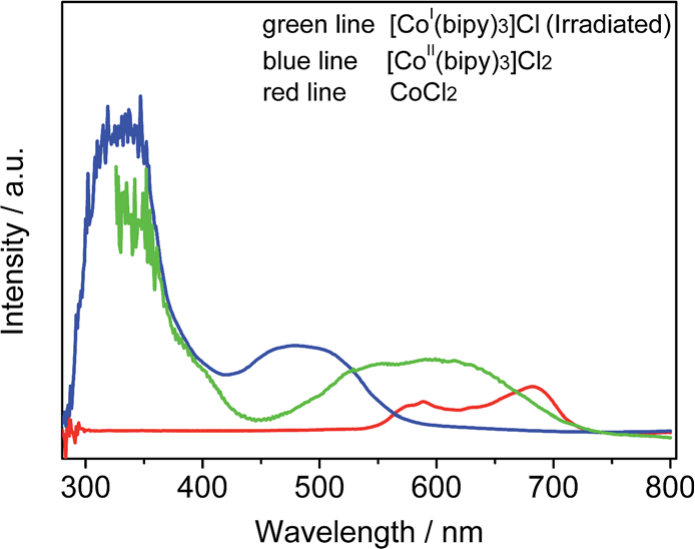
Absorption spectra of acetonitrile solutions in the forms of [Co^I^(bipy)_3_]Cl (green line, containing TiO_2_ spheres), [Co^II^(bipy)_3_]Cl_2_ (blue line) and CoCl_2_ (red line).

To identify the formation of cobalt species, the optical absorption spectra of several solutions including Co^2+^, [Co^II^(bipy)_3_]Cl_2_, and [Co^I^(bipy)_3_]Cl are presented in Fig. 5. The solution containing Co^2+^ exhibited an absorption band positioned at 550–720 nm (red line), which is due to the d–d transition in CoL_4_ tetrahedral complexes.^22^ After the introduction of bipy, a new absorption peak at 400–570 nm was observed in the UV-vis spectrum (blue line), which was in accord with results for Co(bipy)_3_Cl_2_ in previous literature.^23^ These results are due to the formation of a new photo-responsive compound [Co(bipy)_3_]^2+^ when Co^2+^ and bipy were combined. To investigate the effect upon Co^II^ when catalyzed by sTiO_2_, the reaction solution was measured *via* UV-Vis absorption spectroscopy after 10 min of light irradiation (green line). The formation of a new absorption band (*ca.* 450–720 nm) was observed in the UV-Vis absorption spectrum. The broad absorption band is attributed to the intramolecular charge-transfer from the metal center to the pyridinium moiety. This change is mainly due to the generation of a reducing Co^I^-complex during the photocatalytic CO_2_ reduction reaction.

**Fig. 6:**
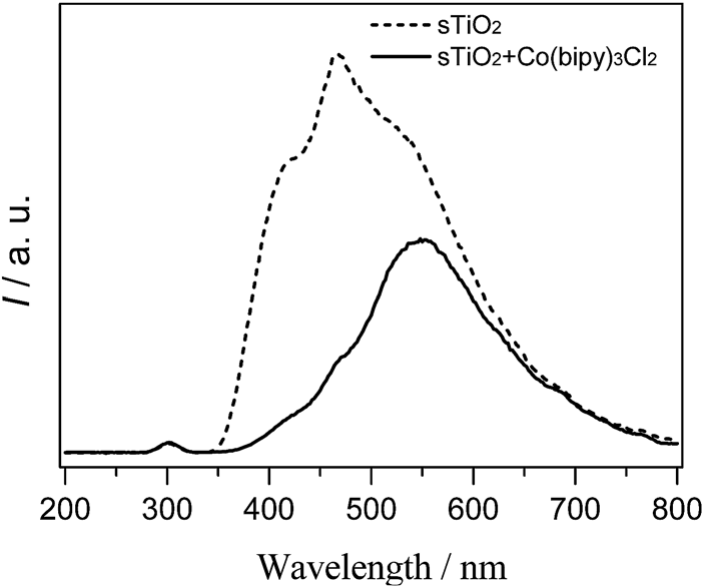
Photoluminescence spectra of semiconductor photocatalysts without (dashed line) and with (solid line) [Co(bipy)_3_]^2+^ .

To determine whether the cobalt complex accelerates charge carrier transfer, we used a PL technique to investigate the fluorescence, and the results are shown in Fig. 6. The sTiO_2_ exhibited a strong PL band that was assigned to the inherent recombination of photogenerated electrons and holes.^24^ In the presence of [Co(bipy)_3_]^2+^, there is a significant decrease in the PL intensity. A weaker intensity of the PL peak represents a lower recombination probability of photo-generated charge carriers. Therefore, the integration of cobalt redox mediators with sTiO_2_ could effectively inhibit the recombination of photogenerated charge carriers and accelerate electron migration.

**Fig. 7:**
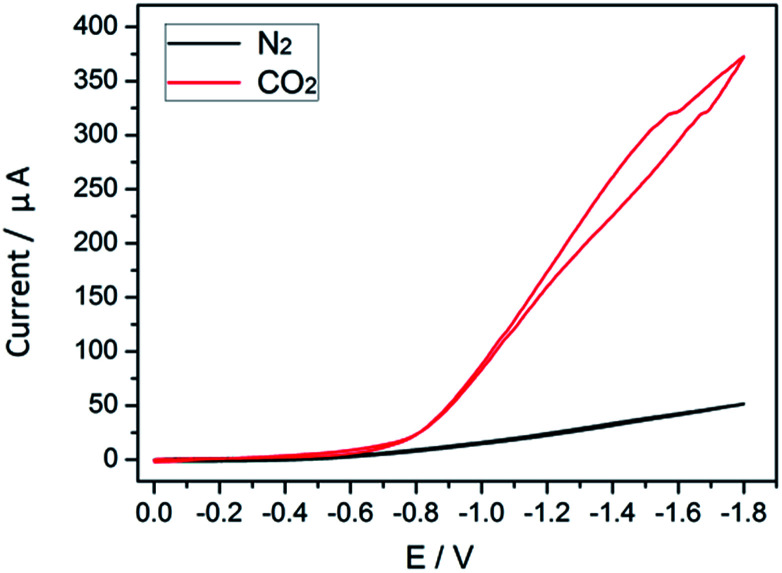
Cyclic voltammograms in N_2_ (black) and CO_2_ (red) saturated solutions.

The process of cobalt complex transformation during CO_2_ reduction can be inspected *via* cyclic voltammetry (CV). In electrochemical tests, the transition state of the cobalt species can be monitored indirectly, which involves the initial reduction of Co^II^ complexes and their subsequent reaction with CO_2_ to form Co–CO_2_ intermediates.^25^ In the absence of CO_2_, the obtained redox potential (*E*_0_ = −0.8 V) in MeCN solution is attributed to the conversion of the Co^II^ complex to Co^I^ species.^26^ When CO_2_ is introduced (Fig. 7), the intensity of the peak observed in N_2_ is enhanced considerably. The markedly increased current densities reveal that the reaction system undergoes the electrocatalytic CO_2_ reduction reaction with high efficiency.

The advantage of hollow spherical TiO_2_ has been demonstrated in the photocatalytic performance of CO_2_ reduction (Fig. 4). We suggest that the hierarchical structure plays a important role in the current reaction. As revealed in Fig. 2, the ultrathin nature of the TiO_2_ nanosheets allows charge carriers to move rapidly onto the surface. In addition, the sufficient separation of the ultrathin TiO_2_ nanosheets enables photogenerated electrons to quickly transfer from the TiO_2_ nanosheets to the cobalt complex to enhance utilization of the charge carriers. With respect to the photoresponse, the hollow structure potentially acts as a photon trap well to allow the multiscattering of incident light for the enhancement of light absorption.

Upon irradiation, the excited TiO_2_ liberates the electrons from the valence band to the conduction band.^27^ CO_2_ activation happens in the absence of [Co(bipy)_3_]^2+^ through charge transfer from the semiconductor to the CO_2_ molecule adsorbed on the TiO_2_ surface. While the presence of massive photogenerated electron–hole pairs is in vain because of the fast recombination, especially in the heterogeneous photocatalytic system, as expected, there is a dominant pathway that is designed for electron transfer towards the CO_2_ molecule *via* the cobalt complex.^28^ After Co^II^ gains an electron, the low valent Co intermediates are stabilized by pyridine ligands through weak coordination,^29^ promoting the activation of the CO_2_ molecules *via* nucleophilic attack. The thus formed Co^I^ complexes are important redox active catalysts.^16^ In our previous work, evidence of the generation of Co^I^ through light excitation was observed *via* UV-vis spectroscopy.^30^ Such intermediates may also result in the protonation of the Co^I^–H complex when hydron is present, subsequently yielding H_2_.^31^ It should be noted that the activity of the CO_2_ conversion reaction is determined not only by the rate of formation of the photogenerated charge carrier, but also the energy barrier to formation of the relevant CO_2_ intermediates. The activation energy of molecular CO_2_ is lowered after the combination of CO_2_ with cobalt species. Thus, CO_2_ reduction exhibits superior competitiveness towards H_2_ generation. As discussed, the following processes are revealed by the electrochemical performance, and are described in eqn (1)–(7).

1 TiO_2_ + *hν* = e^−^ + h^+^

In N_2_-saturated solution

2 Co(ii) + e^−^ → Co(i)

3 Co(i) − e^−^ → Co(ii)

In CO_2_-saturated solution

4 Co(ii) + e^−^ → Co(i)

5 Co(i) + CO_2_ ↔ CO_2_Co(i)

6 CO_2_Co(i) + H^+^ + e^−^ → (CO_2_–)(H–)Co(i)

7 (CO_2_)(H)Co(i) + H^+^ → Co(ii) + CO + H_2_O

## 4 Conclusions

In summary, we have explored improving the efficiency of photocatalytic CO_2_ reduction with TiO_2_ hollow spheres as photocatalysts in synergetic combination with [Co(bipy)_3_]Cl_2_. This hybrid system exhibits high photocatalytic reactivity towards CO_2_-to-CO conversion due to the addition of [Co(bipy)_3_]^2+^ as a promoter for charge-carrier separation, transfer kinetics, and interface interaction. This result provides insight to the construction of efficient heterogeneous photochemical CO_2_ reduction systems under mild conditions. This finding has the potential to open up new opportunities in artificial photosynthesis, catalytic chemistry, and energy conversion with excellent morphology catalytic materials.

## Conflicts of interest

There are no conflicts to declare.

## Supplementary Material

RA-008-C8RA03211D-s001
