# Supplementary material for: Improving the photocatalytic reduction of CO2 to CO for TiO2 hollow spheres through hybridization with a cobalt complex
Source: RSC Adv. 2018 Jun 5;8(37):20543–8. doi: 10.1039/c8ra03211d (PMC9080812; doi:10.1039/c8ra03211d)
Supplement: RA-008-C8RA03211D-s001 [file RA-008-C8RA03211D-s001.pdf]

Electronic Supplementary Material (ESI) for RSC Advances.
This journal is © The Royal Society of Chemistry 2018

Supporting Information

Improving the Photocatalytic Reduction of CO_2 to CO for TiO_2Hollow Sphere through Hybridization with Cobalt Complex

Jinliang Lin[a] *, Xiaoxiang Sun [b] , Biao Qin[a] and Ting Yu[a]

[a] Department of Chemical and Engineering, Zunyi Normal College, 563000, Zunyi, P.R. China.
[b] *Department of Chemical and Engineering, Qiannan Normal University for Nationalities, 558000, DuYun, P.R. China.*
Email:jinliang_lin@163.com

Contents

Fig. S1 SEM images of $bTiO_2$ and Energy-dispersive X-ray spectroscopy (EDS) of the as-prepared $bTiO_2$.
Fig. S2 Mapping of the as-prepared $bTiO_2$.
Fig. S3 SEM images of $sTiO_2$ and Energy-dispersive X-ray spectroscopy (EDS) of the as-prepared $sTiO_2$.
Fig. S4 Mapping of the as-prepared $sTiO_2$.
Fig. S5 SEM images of $sTiO_2$ sample after reaction and Energy-dispersive X-ray spectroscopy (EDS) of the as-prepared $sTiO_2$ sample after reaction.
Fig. S6 Mapping of the as-prepared $sTiO_2$ sample after reaction.
Table S1 Element contents in $bTiO_2$ sample.
Table S2 Element contents in $sTiO_2$ sample.
Table S3 Element contents in $sTiO_2$ sample after reaction.

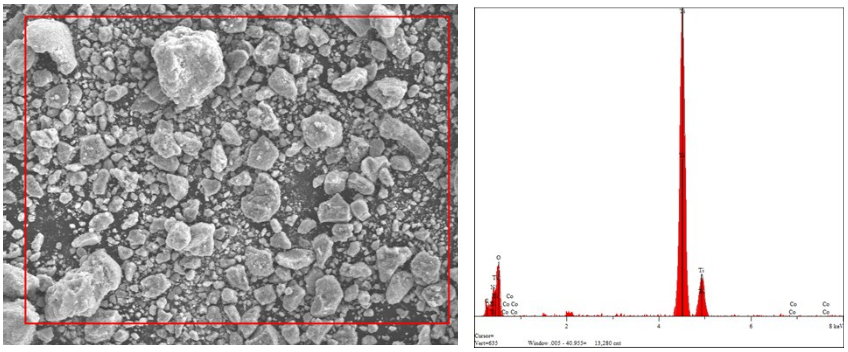

Fig. S1 SEM images of $bTiO_2$(left) and Energy-dispersive X-ray spectroscopy (EDS) of the as-prepared $bTiO_2$(right).

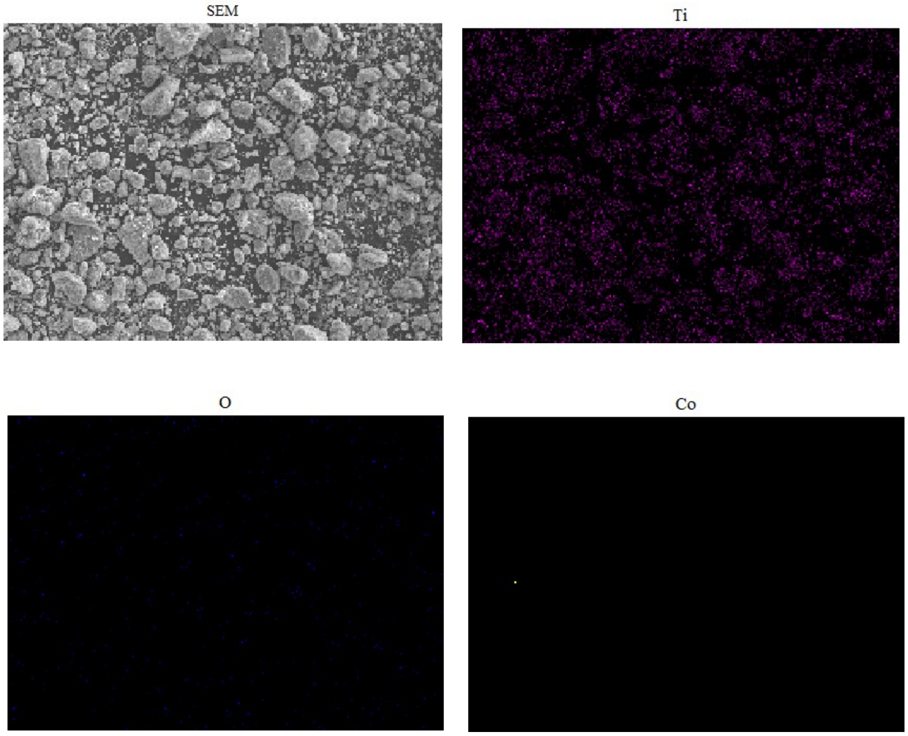

Fig. S2 Mapping of the as-prepared $bTiO_2$.

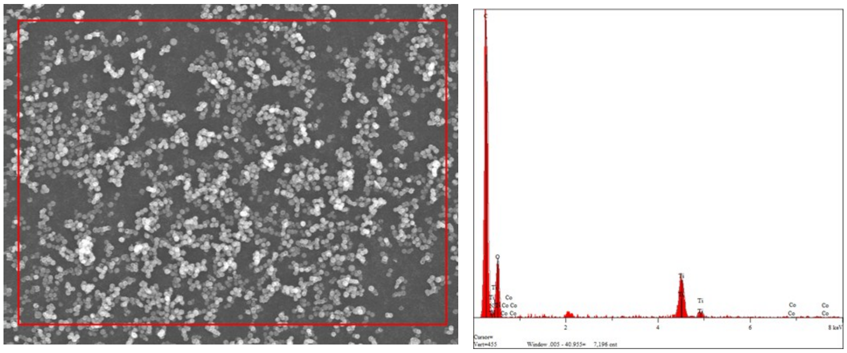

Fig. S3 SEM images of $sTiO_2$(left) and Energy-dispersive X-ray spectroscopy (EDS) of the as-prepared $sTiO_2$(right).

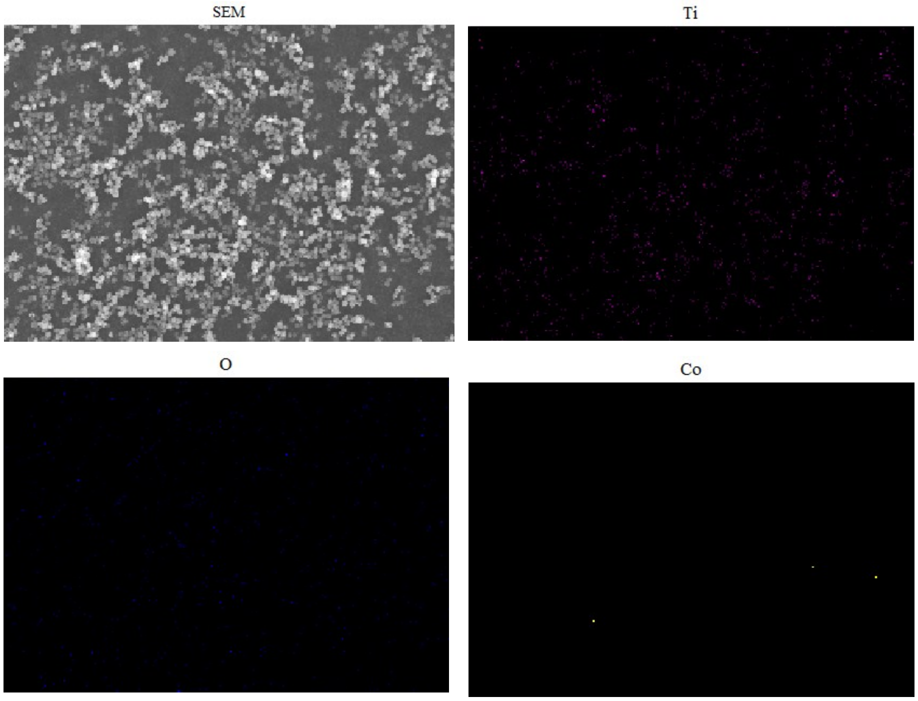

Fig. S4 Mapping of the as-prepared $sTiO_2$.

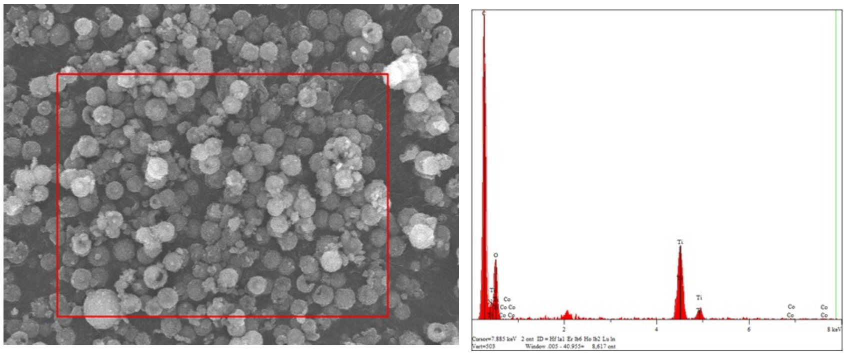

Fig. S5 SEM images of $sTiO_2$ sample after reaction(left) and Energy-dispersive X-ray spectroscopy (EDS) of the as-prepared $sTiO_2$ sample after reaction (right).

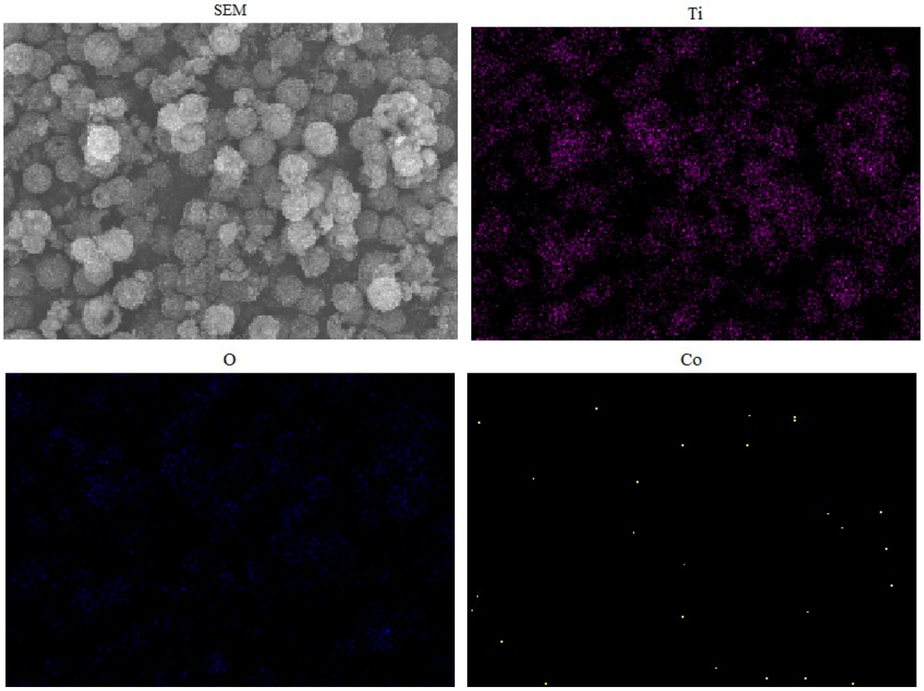

Fig. S6 Mapping of the as-prepared $sTiO_2$ sample after reaction.

Table S1 Element contents in $bTiO_2$ sample.

Elt.	Line	Intensity (c/s)	Conc	Units	Error 2-sig	MDL 3-sig	
C	Ka	28.77	4.982	wt.%	0.852	0.945	
N	Ka	0.27	0.226	wt.%	0.612	0.708	
O	Ka	42.95	28.825	wt.%	3.138	2.402	
Ti	Ka	876.42	65.205	wt.%	1.417	.394	
Co	Ka	0.38	0.062	wt.%	0.344	0.517	
			100.000	wt.%			Total

Table S2 Element contents in $sTiO_2$ sample.

Elt.	Line	Intensity (c/s)	Conc	Units	Error 2-sig	MDL 3-sig	
C	Ka	371.37	54.036	wt.%	1.838	0.742	
N	Ka	0.00	0.000	wt.%	0.000	0.000	
O	Ka	68.87	36.816	wt.%	3.014	1.684	
Ti	Ka	86.61	8.823	wt.%	0.630	0.296	
Co	Ka	1.53	0.325	wt.%	0.305	0.391	
			100.000	wt.%			Total

Table S3 Element contents in $sTiO_2$ sample after reaction.

Elt.	Line	Intensity (c/s)	Conc	Units	Error 2-sig	MDL 3-sig	
C	Ka	384.93	52.819	wt.%	1.722	0.403	
N	Ka	0.00	0.000	wt.%	0.000	0.000	
O	Ka	56.53	31.677	wt.%	2.801	1.328	
Ti	Ka	165.14	15.261	wt.%	0.771	0.268	
Co	Ka	1.25	0.243	wt.%	0.348	0.490	
			100.000	wt.%			Total
